# Chronic obstructive pulmonary disease prevalence and associated factors in an urban HIV clinic in a low income country

**DOI:** 10.1371/journal.pone.0256121

**Published:** 2021-08-13

**Authors:** Ahmed Ddungu, Fred C. Semitala, Barbara Castelnuovo, Christine Sekaggya-Wiltshire, William Worodria, Bruce J. Kirenga

**Affiliations:** 1 Infectious Diseases Institute, Makerere University, Kamplala, Uganda; 2 Makerere University Lung Institute, Kamplala, Uganda; 3 Makerere University College of Health Sciences, Kamplala, Uganda; 4 Makerere University Joint AIDS program, Kamplala, Uganda; 5 Mulago National Referral Hospital, Kamplala, Uganda; Massachusetts General Hospital, UNITED STATES

## Abstract

**Introduction:**

In the last decade, survival of people living with HIV (PLHIV) has dramatically increased due wide availability of effective antiretroviral therapy. However, PLHIV remain at a comparatively higher risk of non-communicable comorbidities. We sought to determine the burden of COPD and its associations in an urban tertiary HIV clinic in Uganda.

**Methods and findings:**

HIV-infected adults attending the Makerere University Joint AIDS program; aged ≥30 years without acute ailments were screened for COPD using study questionnaires and spirometry (post-bronchodilator FEV_1_/FVC<0.7). We determined its prevalence and association with demographic characteristics, body mass index (BMI) and known risk factors.

Of 288 participants enrolled, 177 (61%) were female; 253 (88%) were from urban residences, median age was 45 years (IQR: 39–51), 71(25%) were ‘ever’ smokers, 284(99%) reported biomass fuel use and 72(25%) had a history of tuberculosis. All except 1 participant were on antiretroviral therapy, median current CD4 (cells/mm3) was 558 (IQR 402–753) and 275(96%) were virologically suppressed. Nearly half (130/288, 45%) had recurrent respiratory symptoms. The prevalence of COPD was 3.1% (9/288) [95% CI: 1.63–5.92]. COPD was associated with: previous tuberculosis, (adjusted odds ratio (AOR): 6.36, [95% CI 1.64–35.84], P = 0.036), self-reported chronic shortness of breath (AOR: 9.06, [95% CI 1.34–61.10], P = 0.024) and a BMI <21 Kg/m^2^ (AOR: 10.42 [95% CI: 1.61–100.00], P = 0.013).

**Conclusion:**

In this HIV population, COPD prevalence was low and was associated with previous tuberculosis, self-reported chronic shortness of breath and BMI <21 Kg/m^2^.

## Introduction

By 2017, chronic obstructive pulmonary disease (COPD) had risen to the 3^rd^ leading cause of death worldwide [1,2]. The general COPD prevalence continues to grow including in Africa where it ranges between 4 to 25% [3,4]. On the contrary, global mortality due to HIV continues to fall mainly due to wide availability of combination antiretroviral therapy (ART). In 2019, HIV was the 19^th^ global cause of death (WHO Global Health Estimates). While survival of people living with HIV (PLHIV) has dramatically increased to levels comparable to those of HIV uninfected persons in some populations [5]; PLHIV remain at a comparatively higher risk of non-communicable comorbidities [6]. Earlier studies demonstrated an increased risk of COPD amongst both ART-exposed and ART-naïve PLHIV [7–13]. A recent systematic review and meta-analysis of 30 studies reported a global COPD prevalence of 10.5% among PLHIV; pooled odds ratio (OR) of 1·14, 95% CI 1·05–1·25 (based on 11 studies comparing PLHIV with HIV uninfected persons) and an OR of 2·58, 95% CI 1·05–6·35 after adjustment for tobacco consumption (based on 4 studies) [14]. However, of the 30 studies; only 4 were from Africa and 26 from the Americas, Europe and Japan, regions with significant differences on account of established COPD risk factors. Notably, the former have higher smoking rates but lesser HIV burden compared to sub-Saharan Africa which accounts for 70% of the global HIV burden; a quarter of the global Tuberculosis (TB) burden and very high biomass use. In Uganda, a low income country with an estimated 1.2million PLHIV (HIV prevalence of 5.9%) [15], a few studies have assessed the prevalence of COPD in the general population. It varies between 2–16% in rural Uganda [16–18] and estimated at 1.5% in urban Uganda [17]. However, only one study has assessed the burden of COPD in an HIV care program. Kayongo, Alex, et al. found a prevalence of COPD of 6.2% amongst PLHIV attending four HIV treatment centers in rural Nakaseke district in Central Uganda [19]. This study was published after the above systematic review was conducted. Our aim was to determine the prevalence of COPD and associated factors in a large urban HIV outpatient clinic in Kampala, Uganda.

## Methods

### Study setting and design

This was a cross sectional study aimed at determining the prevalence of COPD and associated factors among PLHIV attending the Makerere University Joint AIDS program clinic, a tertiary HIV out-patient facility at Mulago National Referral Hospital. The facility provides care to a predominantly urban population of over 15,000 adults, mainly from Kampala district, the capital city of Uganda and surrounding metropolitan districts of Wakiso and Mukono. Between 15th October 2016 and 15^th^ Jan 2017 HIV infected adults aged ≥ 30 years and free from acute illness were considered for inclusion in the study. Enrolment was set on 3 week days (Monday, Tuesday and Thursday) known to have the highest patient attendance in the week. Enrolment was done consecutively at the triage station from 8am to include 15–20 participants per day. Patients were excluded if spirometry was contraindicated (e.g. a history of acute cardiac/respiratory illness, active TB or pregnancy). Additionally, severely sick patients requiring admission or with a Karnofsky score < 80% were excluded.

### Data collection

Physical parameters including weight, height, blood pressure, Karnofsky score were measured and the body mass index (BMI) calculated. A pretested study questionnaire adapted from the FRESH AIR survey on COPD in western Uganda [16] was used to collect data on participants’ characteristics. These included: social-demographic (e.g. age, sex, address), chronic respiratory symptoms including chronic cough, chronic sputum production, lifetime experience of a wheeze, and self-reported shortness of breath (effort tolerance) graded using the Medical Research Council (MRC) dyspnoea scale [20]. Risk factors for COPD were also assessed, and included smoking, exposure to biomass fuel (firewood, charcoal) and history of respiratory infections including TB. A smoker was anyone with consumption >100 cigarettes in a life time and former smoker was a smoker who ceased ≥4 weeks before enrolment. HIV specific history was extracted from the clinic electronic database including duration since diagnosis and ART initiation, baseline CD4+ cell count, opportunistic infections, WHO clinical stage and the most recent viral load. As a tertiary HIV facility, intervention is done for all patients with detectable HIV viremia even if its lower than the Uganda nationally set cut off of <1000 copies/ml. We set our study viral suppression cut off at <75 copies/ml. Other laboratory evaluations included complete blood counts and CD4+ cell counts.

### Procedure for spirometry and diagnosis of COPD

Spirometry was performed by a qualified technician using a Vitalograph Pneumotrac desktop spirometer model 6800 as per standard guidelines [21]. COPD was diagnosed using a fixed post-bronchodilator ratio of forced expiratory volume in 1 second (FEV_1_) to forced vital capacity (FVC) ratio of <0.7 using the criteria recommended by the Global Initiative for Chronic Obstructive Lung Disease (GOLD) guidelines [22] and severity graded using the 4 stage classification per same guidelines. A certified spirometry provider from the Makerere University Lung Institute reviewed each test result for quality control using flow and volume time curves to assess for acceptability and reproducibility.

### Ethics and consent to participate

The study was approved by the Makerere University School of Medicine Research Ethics Committee (SOMREC), Makerere University College of Health Sciences (#REC REF 2016–093). Participation was voluntary and a written informed consent was obtained from all participants.

### Statistical analysis

A minimum sample size of 222 participants was determined using the Leslie Kish (1964) formula assuming a 15.4% prevalence based on in a similar setting in Nigeria [23], a type 1 error of 5% and power of 80%. Data was collected using EPI-DATA software (version 3.1) and analysed using STATA (version 12.0). Continuous variables were expressed as medians and means. Normally distributed variables were compared using t-tests, and non-normally distributed variables compared using Wilcoxon rank sum tests. Nominal variables were expressed as frequencies and compared using chi-square test. The level of significance was set at P-value less than 0.05. Determinants of COPD were determined by logistic regression analysis, factors which had a P value of <0.20 at bivariate level where considered for multivariate analysis.

## Results

### General characteristics of study participants

During the study period, 310 participants were screened. Of these, 22 were excluded due to: contraindications to spirometry (04), failure to perform spirometry (06) and uninterpretable spirometry results [12]. The remaining 288 participants were included in the final data analysis. Of these, 177 (61%) were female, 253 (88%) were from urban areas of Kampala, Wakiso and Mukono districts in Uganda, overall median age was 45 years (IQR: 39–51), 63 (22%) were former smokers and 72 (25%) were former TB patients. One hundred ninety-six (68%) had lived with HIV for >5years, 101(35%) had a baseline CD4 count of <200cells/mm^3^ and the mean and median current CD4 (cells/mm^3^) of all participants was 581 (SD 250) and 558 (IQR 402–753) respectively. All study participants except one (>99%) were on combination antiretroviral therapy (ART), with 275(96%) virologically suppressed at <75 copies/ml based on their most recent annual viral load result.

### Prevalence and severity of COPD

Of the 288 participants included, 13 were found to have a FEV1/FVC ratio of <0.7 and underwent post bronchodilator spirometry after which 9 (3.1%) were found to meet the criteria for COPD. Of these; six (06) were GOLD stage 1 while three (03) were GOLD stage 2. Regarding effort tolerance of the 09 subjects with COPD; four were MRC grade 1, three were MRC grade 2 on the MRC dyspnoea scale [20] while 2 reported no form of shortness of breath.

### Symptoms and factors associated with COPD

Overall 130 (45%) participants reported at least one chronic respiratory symptom as follows: cough 86(30%), sputum production 60(21%), shortness of breath 74(26%) and wheezing—60(21%). At univariate analysis (Table 1), COPD was associated with self-reported shortness of breath (P = 0.003), previous TB (P = 0.003), post-TB treatment duration of at least 5 years (P = 0.043) and a low BMI (<21kg/m^2^) (P = 0.011).

**Table 1:** Univariate analysis of characteristics of HIV infected patients attending Mulago Hospital based on COPD status.

Variable	No COPD n = 279(%)	COPD n = 9(%)	OR(95%CI)	P-value
**Sex**				
Male	106(38)	5(56)	1	
Female	173(62)	4(44)	0.49(0.13–1.86)	0.296
**Nature of Address**				
Urban	245(88)	8(89)	1	
Rural	34(12)	1(11)	1.11(0.13–9.15)	0.923
**Chronic cough**				
No	197(71)	5(56)	1	
Yes	82(29)	4(44)	1.92(0.50–7.33)	0.339
**Chronic shortness of breaths**				
No	212(76)	2(22)	1	
Yes	67(24)	7(78)	11.07(2.25–54.60)	**0.003**
**Lifetime wheeze**				
No	223(80)	5(56)	1	
Yes	56(20)	4(44)	3.19(0.83–12.25)	0.092
**Smoking history**				
Never smoked	212(76)	5(56)	1	
Former Smoker	67(24)	4(44)	2.53(0.66–9.70)	0.175
**Exposure to biomass**				
No	12(4)	0(0)	1	
Yes	267(96)	9(100)	-	-
**How often have a chest infection**				
<2 times per year.	183(66)	3(33)	1	
≥2 times a year	96(34)	6(67)	3.81(0.93–15.58)	0.062
**Previous TB**				
No	214(77)	2(22)	1	
Yes	65(23)	7(78)	11.5(2.33–56.83)	**0.003**
**Duration since TB treatment**				
≤5 years	43(71)	2(29)	1	
>5 years	18(30)	5(71)	5.97(1.05–33.67)	**0.043**
**Duration since HIV diagnosis**				
≤5years	90(98)	2(2)	1	
>5years	189(96)	7(4)	1.67 (0.33–8.18)	0.529
**Baseline CD4 count (cells/μL)**				
≥200	183(66)	4(44)	1	
<200	96(34)	5(56)	2.38(0.63–9.08)	0.203
**Duration on ART**				
>24 months	237(86)	8(89)	1	
≤24 months	38(14)	1(11)	1.28(0.16 -10.55)	0.817
**Current HIV WHO clinical stage**				
stage1	80(29)	1(11)	1	
stage2	109(39)	0(0)	-	
stage3	75(27)	8(89)	8.55(1.04–69.86)	**0.046**
stage4	15(5)	0(0)	-	
**Spirometry findings**				
FEV1[pre-bronchodilator]mean(SD)	2.605[0.038]	1.903(0.200)		**0.0014**
FEV1%predicted [pre-bronchodilator] mean (SD)	105.71[0.966]	76.11(5.040)		**<0.001**
FVC [pre-bronchodilator]	-	-		
**BMI**				
≥21	229(82)	4(44)	1	
<21	50(18)	5(56)	5.72(1.48–22.08)	**0.011**

Abbreviations: COPD: Chronic obstructive pulmonary disease, FEV1: Forced expiratory volume in 1 second, FVC; Forced vital capacity, ART: antiretroviral therapy, SD: Standard deviation, TB: Tuberculosis, HIV: Human immunodeficiency virus, WHO: World Health Organisation, BMI: Body mass index.

In multivariable analysis (Table 2), factors independently associated with COPD included: chronic shortness of breath, adjusted odds ratio (AOR) 9.062, 95% CI 1.34–61.10, P = 0.024, previous TB, AOR 6.355, 95% CI 1.12–35.84, P = 0.036, and BMI < 21kg/m^2^, AOR 10.416 95% CI 1.61–100.0 P = 0.013.

**Table 2:** Factors associated with COPD among HIV infected participants attending Mulago Hospital at multivariate analysis.

	Adjusted Odds Ratios		
	OR	95% CI	P-value
**Chronic shortness of breaths**	9.062	1.34–61.10	**0.024**
**Life time wheeze**	2.136	0.33–13.62	0.422
**Smoking status**			
Never smoker	Reference		
Ever smoker (current & former)	1.199	0.21–6.84	0.838
≥**2 chest infections per year**	2.904	0.57–14.78	0.199
**Previous TB**	6.355	1.12–35.84	**0.036**
**BMI (kg/m** ^**2**^ **)**			
≥21	Reference		
<21	10.416	1.61–100.0	**0.013**

Abbreviations: COPD: Chronic obstructive pulmonary disease, ART: Antiretroviral therapy, TB: Tuberculosis, HIV: Human immunodeficiency virus, CD4: Cluster of differentiation 4, BMI: Body mass.

### Previous TB and COPD

Seventy-two (10%, n = 288) participants reported previous TB treatment. Seven (10%, n = 72) of these post-TB participants were found with COPD compared to two (<1%, n = 216) among those with no history of TB treatment (P<0.001). Age among participants with and without history of TB was comparable (mean, 46 versus 45 years respectively, P = 0.777). However, the former had a lower BMI (P = 0.030), more chronic respiratory symptoms (i.e. dyspnoea- P = 0.02, wheezing- P = 0.044), more chest infections per year (P = 0.044) and poorer pulmonary function (see Table 3) though no difference in smoking habits when compared to the rest of the other study participants. Other characteristics are compared in Table 3 below.

**Table 3:** Comparison between ‘never treated’ for TB and post-TB treated participants.

	Never-TB N = 216(%)	Post-TB N = 72(%)	p-value
COPD occurrence	2 (1)	7(10)	<0.001
Age, mean [SD]	46 [0.67]	45 [1.00]	0.774
Chronic cough	60 (28)	26 (36)	0.181
Sputum	42 (19)	18 (25)	0.315
Shortness of breath	48 (22)	26 (36)	0.020
Wheezing	39 (18)	21 (29)	0.044
Duration since HIV diagnosis			0.491
≤ 5years	127 (59)	39 (54)	
> 5years	89 (41)	33 (46)	
Baseline CD4, mean [SD]	314.06 [212.23]	260.13 [217.36]	0.064
Current CD4, mean [SD]	592.42 [248.90]	546.67 [249.28]	0.179
BMI (kg/m^2^)			0.030
< 21	35 (16)	20 (28)	
≥ 21	181 (84)	52 (72)	
Former smoker	52 (24)	19 (26)	0.693
Pre-bronchodilator FEV1(L), mean [SD]	2.61 [0.62]	2.48 [0.73]	0.080
% predicted pre-bronchodilator FEV1, mean[SD]	106.29 [16.10]	100.16 [17.99]	0.004
FEV1/FVC ratio [SD]	0.81 [0.07]	0.78 [0.09]	0.006
≥2 Chest infections in a year	39 (18)	21 (29)	0.044

COPD: Chronic pulmonary obstructive pulmonary disease, TB: Tuberculosis, SD: Standard deviation, FEV1: Forced expiratory volume in 1 second, FVC: Forced vital capacity, L-litres, BMI: Body mass index, CD4: Cluster of differentiation 4.

## Discussion

In this study we aimed to determine the burden and associated factors of COPD among PLHIV in an urban HIV clinic in Uganda. We found the prevalence of COPD at 3.1% using the GOLD criteria. The main factors associated with COPD in this population were previous TB, self-reported shortness of breath and low BMI (<21Kg/m^2^). We did not find any association between COPD and urban or rural residence status, smoking, baseline or current CD4 count or duration since HIV diagnosis. Nearly all study participants (99%) were chronically exposed to biomass fuel and hence association between COPD status and biomass use could not be determined.

To our knowledge, this is the first study reporting the prevalence of COPD in a large urban tertiary HIV care program in Uganda. With nearly all study participants virologically suppressed (96%) and with a mean current CD4 cell >500cells/mm^3^; our results indicate effective HIV control in our study population. This may be expected in an urban tertiary HIV facility in a PEPFAR funded country in Africa where effective ART is widely available and free. However, in spite of well-controlled HIV, we found a large burden of chronic respiratory symptoms among study participants (cough 30%, shortness of breath 26%, wheeze 21%, sputum production 21%). Whereas 45% of the participants had at least one chronic respiratory symptom, only self-reported ‘shortness of breath’ was associated with COPD. Earlier studies in the HAART era reported a high symptom burden [8,24]. A recent systematic review and meta-analysis also concluded that PLHIV are more likely to experience respiratory symptoms compared to HIV negative controls [25]. While ART access reduced this association; it remained significant regarding shortness of breath, OR 1.39 (95% CI 1.11 to 1.73). However, most data included in the above systematic review were from developed countries where smoking rates are comparatively higher than in low income Africa. In Uganda, a population based asthma survey in 2016 also showed a fairly high symptom burden among PLHIV (cough 23%, shortness of breath 18%, wheeze 11%) [26]. Like in our study, Pefura et al. also reported a high respiratory symptom burden among PLHIV (47%) in urban Cameroon [27]. However, in contrast to the above, a recent study assessing respiratory symptoms burden by HIV status in urban South Africa, found a low burden and no difference by HIV status (except for breathlessness) [28]. Importantly though, almost all participants routinely used gas or electricity for cooking and heating (99.5%) and not biomass, the default in most low income Africa. Such a study could highlight on the role of high biomass exposure and occurrence of chronic respiratory symptoms in sub-Saharan Africa contexts with low levels of smoking. In the general population, chronic respiratory symptoms are associated with poorer health related quality of life even without asthma or COPD [29]. This may be similar amongst PLHIV who are already battling a chronic infection. Even though screening for chronic respiratory symptoms and diagnostic lung function testing of symptomatic individuals is not routine in HIV programs especially in low income countries like Uganda.

Our results are comparable to a few studies in the context of PLHIV in sub-Saharan Africa. Kayongo, Alex, et al. conducted a similar study that assessed the burden and determinants of COPD amongst 722 PLHIV attending 4 HIV treatment centres in rural central Uganda [19]. Together with our study, the two studies allow us an opportunity to characterize COPD among PLHIV in both rural and urban set ups in Uganda, a low income African country with low smoking rates. Our results reveal a lower prevalence of COPD in the urban PLHIV (3.1%) compared to those in rural Uganda (6.1%). Notable similarities in both studies include: HIV care program based settings with well controlled HIV (>90% HIV virological suppression in both studies, median current CD4 (cells/mm3) of 558, IQR 402–753 and 478, IQR 346–663 in our study and that by Kayongo et al. respectively). Others include young study populations (mean ages 45 and 48 years), predominantly female and never smokers. Of note though, despite a difference in proportion of post-TB patients (25% versus 9.1%), both studies identified TB as a significant predictor of COPD among PLHIV (AOR 6.355, 95% CI 1.12–35.84, in the current study and AOR = 4.92, 95% CI 1.71–14.15, Kayongo et al.). In our study, at univariate analysis, participants with previous TB had a lower BMI, reported more chronic respiratory symptoms, more chest infections per year, and had poorer pulmonary function compared to those without history of TB (Table 3). Differences in prevalence of COPD among PLHIV in urban and rural Uganda may point to differences in exposures to certain COPD risk factors in both settings. For example, early life exposure to biomass and poorer socioeconomic status among rural residents may lead to a higher COPD risk compared to urban residents. Our reported COPD prevalence is however similar to that reported by Pefura-Yone et al. (prevalence of 2.2%) in a case control study in an urban Cameroon [27]. Similarly, in the above study, previous TB was a determinant of COPD in the PLHIV group. In the general population, TB increases the risk of COPD by threefold [30]. One study in South Africa showed incremental risk of chronic lung impairment per episode of TB suffered [31]. Another longitudinal HIV cohort in South Africa showed that a post-TB state was associated with an accelerated annual loss of lung function (35ml and 57 ml excess annual loss of FEV1 and FVC respectively) [32]. However, mechanisms for TB associated airflow obstruction have not been fully elucidated. Suggested pathways include accelerated decline in FEV1 after TB and lung remodelling due to multiple pathological mechanisms including cavitation with distortion of airways, bronchiectasis, bronchogenic/endobronchial TB spread, airway inflammation and narrowing among others [33]. Our results however, together with results by Kayongo Alex et al. and Pefura-Yone et al. underscore the association between post-TB status and COPD even among PLHIV in low income African settings. This is contrary to the widely held view that TB among PLHIV is atypical and associated with minimal residual lung damage due to a compromised immune response. Uganda is among the 30 countries with the highest global TB/HIV burden. This indicates a large population at risk of COPD among PLHIV in Uganda. Both COPD and TB cause chronic cough. However, because TB is the default diagnosis of chronic cough in most HIV care settings in sub-Saharan Africa, many post-TB patients who present with COPD-like exacerbations who test negative on GeneXpert are retreated as clinically diagnosed pulmonary TB relapse, without any consideration of COPD. Therefore, this calls for affirmative action within HIV programs in regard to long term follow up and timely COPD screening among post-TB HIV infected patients with chronic respiratory symptoms.

A low BMI (<21kg/m^2^) was also a key predictor of COPD in our study. We had few patients with COPD and hence the imprecise effect size (AOR 10.416, 95% CI 1.61–100.0 P = 0.013) However, Kayongo et al. also reported an association between COPD and low BMI among PLHIV. COPD patients in that study had a mean BMI of 19.3 kg/m^2^ and each 5 kg/m^2^ increase in BMI reduced the odds of COPD significantly. In Cameroon, Pefura et al. also reported an association between BMI and COPD among PLHIV [27]. Low BMI is associated with mortality among COPD patients in the general population [34]. Whether this extends to HIV infected patients is currently unknown. However, from the above, it is possible that the clinical phenotype of COPD among PLHIV in low income settings like ours is characterised by a low BMI.

However, despite significant similarities in study setting, our results significantly differ from those published by Akanbi et al. who reported a high COPD prevalence of 15.4%, (based on GOLD criteria), among PLHIV in Nigeria [23]. COPD in that study was associated with age > 50 years. We are unable to explain this large disparity. Potential reasons may include difference in population characteristics between East and West Africans, although this appears insufficient to explain the large difference. Differences in study tools (e.g. Akanbi et al. didn’t report on BMI) or in measures of association may prevent direct comparisons but generally participants in that latter study had a long duration lived since HIV diagnosis (mean of 8 years SD2.6), and a low mean baseline CD4 (median 173 cell/mm^3^ IQR 29–283). Our participants had better HIV control compared to those studied by Akanbi et al. (HIV viral suppression of 96% versus 67.2%). In spite of the high COPD burden, Akanbi et al. comparatively reported a lesser burden of chronic respiratory symptoms (17.4% versus 45% in our study), lower burden of biomass exposure (37.9% versus 99% in our study) and smoking (17% versus 25% in our study). Of note though, while our studies had a similar proportion of post-TB patients (25% in our study versus 23.5% in the latter study), Akanbi et al. didn’t find any association between COPD and post-TB status. This is in major contrast with our study and the above studies by Kayongo et al. and Pefura et al. Therefore, there results generally differ from the growing literature on COPD burden and risk factors among PLHIV in the sub-Saharan context and may be point to different COPD risk factors among PLHIV in Nigeria.

Generally, differences in diagnostic criteria pose difficulty in comparing our findings with broader COPD-HIV data as majority of the earlier studies used physician based diagnosis or patient self-report [7] rather than spirometry to diagnose COPD. A recent meta-analysis highlighted a global COPD prevalence of 5.6–10.6% among PLHIV [14], fairly higher than in our study, although most data were from developed countries with higher smoking rates, lower biomass and TB exposure compared to our study setting. Therefore, generalisability of those results to our study setting is difficult.

The strength of our study lies in the high quality of spirometry, which resulted in the exclusion of very few patients from final analysis thereby minimizing measurement bias. Our study had some limitations as follows: We used a single spirometry test to classify COPD, given our cross-sectional study design and hence, may have misclassified COPD status in participants with borderline lung function as shown by some studies [35]. Additionally, we used the GOLD criteria solely to diagnose COPD, this criteria has been shown to underestimate COPD in the young adults, a predominant population in our study. Some similar studies showed a variation of COPD prevalence across different spirometric reference equations [23,36]. We also excluded severely ill patients out of safety concerns for spirometry without additional workup. Some of these may have been patients with severe COPD. We only assessed for post bronchodilator reversibility among participants with FEV/FVC <0.7 and hence may have missed an opportunity to detect asthmatics given the high number of participants with a lifetime wheeze. Lastly, because of a cross-sectional design, we are not able to adduce causality.

## Conclusion

In conclusion, we demonstrate that among PLHIV who are free from acute illness; COPD prevalence is low and is associated with a history of tuberculosis, self-reported shortness of breath and BMI<21 Kg/m^2^.

## Supporting information

S1 File Study questionnaire. (PDF)
