# Supplementary material for: Chronic obstructive pulmonary disease prevalence and associated factors in an urban HIV clinic in a low income country
Source: PLoS One. 2021 Aug 13;16(8):e0256121. doi: 10.1371/journal.pone.0256121 (PMC8362990; doi:10.1371/journal.pone.0256121)
Supplement: S1 File — (PDF) [file pone.0256121.s001.pdf]

Study Questionnaire	
**Socio-demographic data**	
study ID:<br><br>|____|____|____|____|	ISS clinic ID:<br><br>|____|____|____|____|____|____|____|____|
Date:<br><br>|____|____|/|____|____|/|____|____|____|____|	Initials :<br><br>|____|____|____|____|
Sex: ☐Male ☐Female	Tel: /
DOB:<br><br>|____|____/|____|____/|____|____|____|____|	Nature of Address: ☐urban ☐Rural ☐Suburban
Address:<br><br>☐ Kampala ☐Wakiso ☐other (list)……………………………………	
Occupation: ☐Peasant/Farmer ☐Construction & quarries ☐Brick making ☐Flour & grain workers ☐charcoal business ☐Mining ☐Carpentry ☐Textile ☐Petroleum ☐welding ☐ Business (specify)……………….. ☐Professional(specify)……..………... ☐Unemployed ☐other………………..	
Religion:<br>☐Catholic ☐Muslim ☐Anglican ☐Pentecostal ☐ Other (Specify) …………………….	
Highest level of education attained:<br><br>☐Primary school ☐Secondary school ☐Technical/ Vocational ☐University ☐No formal education	
**Eligibility criteria**	

	**Inclusion criteria**	**Include**	**Exclude** (if any is ticked)
1	HIV positive	☐ YES	☐ NO
2	Attending Mulago ISS clinic	☐ YES	☐ NO

3	Aged ≥30years				☐ YES	☐ NO	
4	Able and willing to give written informed consent?				☐ YES	☐ NO	
	**Exclusion criteria(exclude if yes)**	YES	NO			YES	NO
1	Pregnant	☐	☐	5	Unstable angina	☐	☐
2	Recent abdominal surgery	☐	☐	6	Active TB	☐	☐
3	Documented pneumonia within 4wks	☐	☐	7	Current pneumothorax	☐	☐
4	Myocardial infarction within last 4wks	☐	☐	8	Very sick(requires admission)	☐	☐
	All inclusion criteria met and no exclusion: ☐**YES** ☐**NO**						
	If yes; assign study id:<br>|____|____|____|____| *(then Proceed to obtaining informed consent)*						

Date collected by:

|____|____|____| (initials) Date: |____|____|/|____|____|/|____|____|____|____| Time: |____|____|____|____

**Symptoms of chronic obstructive disease:**	
1	Do you cough several times most of the day? ☐YES ☐ NO
2	If yes, when do you cough? ☐In the morning ☐During the day ☐During the night
3	For how long you have been coughing? ☐………Months (≥1 - <12) ☐……..Years
4	Sputum: Do you bring up phlegm or mucus on most days? ☐YES ☐NO
5	Shortness of breath: Do you run out of breath more easily than others of your age? ☐YES ☐NO
6	If yes , describe your exercise tolerance below:<br>☐ 0- I only get breathless on strenuous exercise

	☐ 1- I get short of breath when hurrying on the level or walking up a slight hill<br>☐ 2- I walk slower than people of the same age on the level because of shortness of breath or I have to stop for breath when walking at my own pace on the level<br>☐ 3- I stop for breath after walking about 100 yards or after a few minutes on level ground<br>☐ 4- I am too short of breath to leave the house or I am breathless when dressing or undressing.
7	Wheezing: do you wheeze or have any whistling in the chest? ☐Yes ☐NO
8	When did you first notice wheezing or have whistling in the chest? (onset)<br>☐………Years ago ☐………Months ago ☐………Weeks ago
9	Do you wake up with wheezing? ☐YES ☐NO
10	Have you been at all breathless when wheezing was present? ☐YES ☐NO
11	Have you ever experienced chest tightness when you are wheezing? ☐YES ☐NO
12	Are there some factors triggering your wheezing ☐YES ☐NO
13	If the answer is yes (in 12) specify:…………………………
14	Do you have allergies? ☐Yes ☐NO
15	If answer is yes (in 14), specify……………………………………
16	At what age did you start having allergies? ……………..
17	Do you have family history of asthma? ☐YES ☐NO
18	Have you been told that you suffer from heart disease or heart failure: ☐YES ☐NO
19	If yes (in 18) list current medication:
**RISK FACTORS**	
1	Smoking history: ☐Current smoker ☐Former smoker ☐Never smoked<br>*****smoker: a person who has consumed >100 cigarettes in a life time; a former smoker: one who has consumed >100 lifetime cigarettes but not smoked in ≥4wks***
2	If the answer is current smoker or former smoker:<br>Average number of cigarettes smoked per day……… Smoking duration in years……..or months….....

3	Which products do you use?<br>☐Cigarettes ☐pipe ☐Cannabis ☐other local tobacco products, specify……………………………
**Biomass fuel use**	
1	What do you use for cooking?<br>☐Fire wood ☐Charcoal ☐Electricity/gas ☐Other…………………………
2	How long have you been using this fuel? ....years ………….months
3	Do you have a chimney in your kitchen? ☐Yes ☐NO
4	Do you use kerosene for lighting or cooking? ☐ Yes ☐NO
5	Time spent cooking <u>indoor</u> …..……hours/day
6	Time spent cooking <u>outdoor</u> ………..hours/day
7	Where do you sleep? ☐Same room as kitchen<br>☐Separate room but in the same house as the kitchen ☐ Separate house from the kitchen
8	How many windows does the room you sleep in have? ☐0 ☐1 ☐2 ☐3 ☐>3
**Respiratory symptoms and infections**	
1	How often do you have a chest infection? ☐> 2 per year ☐<1 per year
2	History of childhood respiratory infection ☐Frequent ☐Sometimes ☐Seldom
3	Have you ever been treated for TB? ☐YES ☐NO
4	If yes, period of treatment onset (years ago?): ☐0- 2 ☐>2-5 ☐>5-10 ☐ >10
**HIV history**	
1	When where you first diagnosed with HIV? (years ago) ☐0- 2 ☐>2-5 ☐>5-10 ☐ >10
2	Have your chest symptoms or infection increased after being diagnosed with HIV? ☐YES ☐NO
3	Are you taking ARVs? ☐YES ☐NO if yes, current regimen …………………………
4	Baseline CD4 count |____|____|____|____| Date: |____|____|/|____|____|/|____|____|____|____|
5	Duration on ART? ☐0- 6mo ☐ >6 mo - 1yr ☐>2-5yrs ☐>5-10yrs ☐ >10yrs

6	If yes have your symptoms improved on ART use? ☐YES ☐NO
7	Have you ever been diagnosed with any of the following conditions:<br>☐ PCP/PJP ☐ Pneumonia ☐ Lymphoma ☐ kaposi's sarcoma ☐Cryptococcal meningitis
8	Current HIV WHO clinical stage: ☐ 1 ☐2 ☐3 ☐4

Exacerbations and antibiotics use

1	How many times in the past year did you have a serious chest infection that lasted at least 7 days and caused you to stop of your usual work or gave you a lot of trouble with your usual chores?<br>☐0 (none) ☐ 1-2 times ☐3 or more times
2	How many times in the past year did you get antibiotics for your Chest infection?<br>☐0 (none) ☐1-2 times ☐3 or more times

Physical exam

Weight (kg)	Height (meters)………	BMI:………………	Temperature: ………C
BP(mmHg):……….	Heart rate (bpm)………	SPO2(%)………….	Kanofsky score ……..%

<u>SPIROMETRY</u> Findings

**Pre-bronchodilator findings**	Result	%predicted	**Post- bronchodilator findings**	Result	%predicted
**FEV1**			**FEV1**		
**FVC**			**FVC**		
**FEV1/FVC ratio**			**FEV/FVC ratio**		
			**Reversibility %**		

**Does Patient have COPD? (post bronchodilator FEV1/FVC ratio <0.7)**		☐**YES**		☐**NO**	
If yes, proceed to clinical COPD questionnaire below scale below	**GOLD STAGE**	☐1	☐2	☐3	☐4

CLINICAL COPD QUESTIONNAIRE

Please circle the number of the response that best describes how have you been feeling during the past week

(only one response for each question)								
	On average, during the past week, how often did you feel:	never	Hardly ever	A few times	Several times	Many times	A great many times	Almost all the times
1	Short of breath at rest?	0	1	2	3	4	5	6
2	Short of breath doing physical activity?	0	1	2	3	4	5	6
3	Concerned of getting cold or your breathing getting worse?	0	1	2	3	4	5	6
4	Depressed (down) because of breathing problems?	0	1	2	3	4	5	6
	In general, in the past week how much of the time:							
5	Did you cough?	0	1	2	3	4	5	6
6	Did you cough phlegm?	0	1	2	3	4	5	6
	On average, during the past week, how limited were you in these activities because of your breathing problems?	Not limited at all	Very slightly limited	Slightly limited	Moderately limited	Very limited	Extremely limited	Totally limited/ unable to do
7	Strenuous activities (such as climbing stairs, hurrying, doing sports)?	0	1	2	3	4	5	6
8	Moderate physical activities (such as walking, house work, carrying things)?	0	1	2	3	4	5	6
9	Daily activities (such as dressing and washing yourself)?	0	1	2	3	4	5	6
10	Social activities (such as talking, visiting friends, being with children)?	0	1	2	3	4	5	6

**LABORATORY RESULTS**			
WBC	/µL	CD4+ count □ Yes □ No	cells /µL
ANC	/µL	CD8+ count	cells /µL
LYMPH	/µL	CD4:CD8 ratio	
HB	g/dL	Viral load □ Yes □ No	copies /mL

Appendix IV: Karnofsky Score (Performance Status)		
Functional status	%	characteristics
Able to carry on normal activity, no special care is needed.	100	Normal; no complaints; no evidence of disease.
	90	Able to carry on normal activity; minor signs or symptoms
	80	Normal activity with effort; some signs or symptoms of disease
Unable to work; able to live at home and care for most personal needs; a varying amount of assistance is needed	70	Cares for self; unable to carry on normal activity or to do active work.
	60	Requires occasional assistance but is able to care for most needs.
	50	Requires considerable assistance and frequent medical care.
Unable to care for self; requires equivalent of institutional or hospital care; disease may be progressing rapidly	40	Disabled; requires special care and assistance
	30	Severely disabled; hospitalization is indicated though death not imminent
	20	Very sick; hospitalization necessary; active supportive treatment necessary
	10	Moribund; fatal processes progressing rapidly
	0	Dead.
